# Repurposed cAMP-Modulating Agents Enhance 5-Fluorouracil Response Through Membrane-Dependent Mechanisms

**DOI:** 10.3390/membranes16070217

**Published:** 2026-06-26

**Authors:** Eduarda Ribeiro, Nuno Vale

**Affiliations:** 1 PerMed Research Group, RISE-Health, Faculty of Medicine, University of Porto, Alameda Professor Hernani Monteiro, 4200-319 Porto, Portugal; eduardaprr@gmail.com; 2 ICBAS—School of Medicine and Biomedical Sciences, University of Porto, Rua de Jorge Viterbo Ferreira, 228, 4050-313 Porto, Portugal; 3 RISE-Health, Department of Community Medicine, Health Information and Decision (MEDCIDS), Faculty of Medicine, University of Porto, Rua Doutor Plácido da Costa, 4200-450 Porto, Portugal; 4 Laboratory of Personalized Medicine, Department of Community Medicine, Health Information and Decision (MEDCIDS), Faculty of Medicine, University of Porto, Rua Doutor Plácido da Costa, 4200-450 Porto, Portugal

**Keywords:** 5-fluorouracil, plasma membrane, cAMP signaling, drug repurposing, lipid rafts, membrane transporters, redox homeostasis, chemoresistance, bladder cancer, prostate cancer

## Abstract

Despite its established role as a cornerstone of chemotherapy for solid tumors, 5-Fluorouracil (5-FU) clinical efficacy remains limited by chemoresistance and heterogeneous drug response. Traditional explanations have focused on intracellular metabolism and genetic determinants; however, increasing evidence identifies the plasma membrane as a critical regulatory interface controlling drug availability, signaling integration, and cell fate. Here, we propose a membrane-centered framework in which compartmentalized cAMP/PKA signaling, modulated by repurposed vasoregulatory agents—levosimendan, milrinone, and terbutaline—enhances 5-FU response by functionally remodeling the cancer cell membrane. This remodeling may influence lipid raft organization, ENT1/SLC29A1 transporter trafficking, and the balance between drug influx and efflux, increasing intracellular 5-FU bioavailability and overcoming membrane-mediated pseudo-resistance. In parallel, cAMP-dependent signaling may modulate redox homeostasis, mitochondria-associated membranes, and apoptotic threshold regulation, shifting the cellular response toward irreversible cell death. Importantly, this framework is reconciled with canonical resistance mechanisms—including TYMS upregulation, DPD overexpression, and MMR deficiency—positioning membrane phenotype as a functionally upstream regulatory layer. Differential sensitivity observed experimentally in bladder versus prostate cancer models supports the concept of integrated membrane phenotype biomarkers. Clinical translation requires rigorous pharmacokinetic–pharmacodynamic validation and cardiovascular safety assessment. Redefining the plasma membrane as a dynamic therapeutic interface may provide a rationale for drug repurposing, patient stratification, and personalized combination strategies.

## 1. Introduction

**Figure 1:**
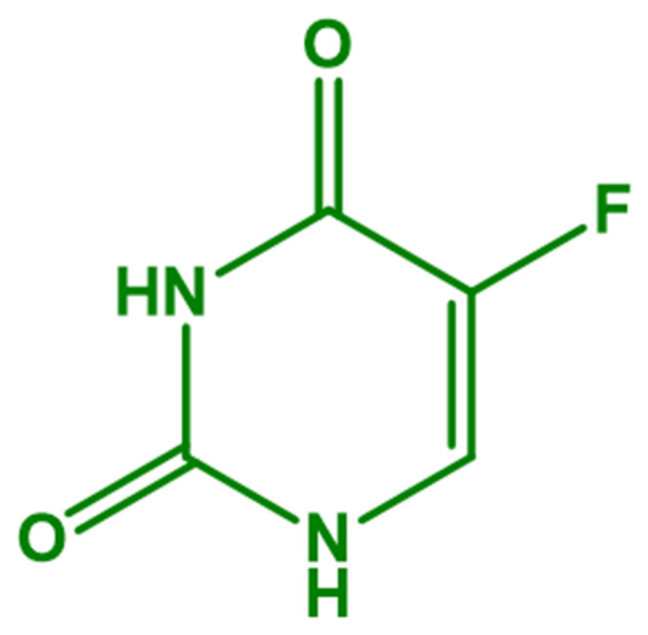
Chemical structure of 5-Fluorouracil (5-FU). Molecular structure of the hydrophilic uracil analogue 5-FU (C_4_H^3^FN_2_O_2_), featuring a fluorine atom substitution at the carbon-5 (C-5) position.

Fluoropyrimidines, such as 5-fluorouracil (5-FU), have stood as a cornerstone of systemic chemotherapy for solid tumors for over six decades [1,2]. Despite their foundational role, clinical outcomes are frequently compromised by the emergence of chemoresistance and significant inter-individual variability in drug response [2,3]. While classical explanations have focused on intracellular metabolism and genetic determinants, increasing evidence points to the plasma membrane as a critical regulator of drug response [4]. As a hydrophilic molecule, 5-FU (Figure 1) relies heavily on specialized membrane transport systems to reach its intracellular targets [5,6].

Consequently, the rate of drug accumulation within the cytosol is strictly regulated by the plasma membrane, where any imbalance between influx and efflux mechanisms can lead to a state of pseudo-resistance, where the drug fails to reach a critical therapeutic concentration despite being present in the extracellular environment [7].

**Figure 2:**
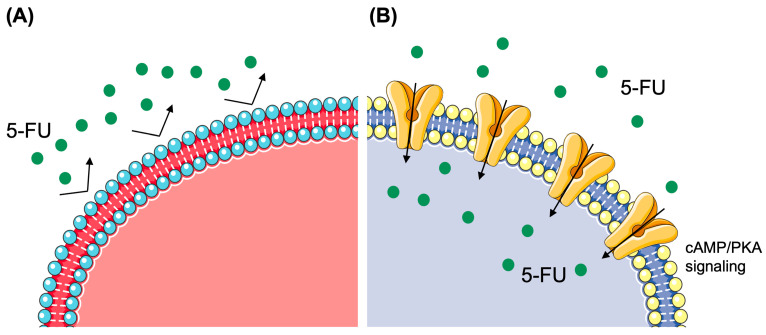
Conceptual model of membrane gating in cancer therapy. (**A**) Under basal conditions, a restrictive and homogeneous membrane interface limits 5-FU entry, leading to a state of pseudo-resistance characterized by low intracellular drug bioavailability. (**B**) Targeted modulation via cardiovascular and vasoregulatory agents remodels the molecular landscape of the cell surface. This cAMP/PKA-dependent signaling “opens the membrane gates” by triggering the translocation of transport proteins (e.g., ENT1) and altering microdomain dynamics, ultimately maximizing 5-Fluorouracil uptake and chemosensitivity. This figure was created using SMART—Servier Medical Art, licensed under CC BY 4.0 (https://creativecommons.org/licenses/by/4.0/). Available online: https://smart.servier.com (accessed on 23 April 2026).

Strategic drug repurposing offers a promising avenue to bypass these transport barriers by modulating the cellular environment and enhancing chemosensitivity. In this context, cardiovascular and vasoregulatory agents—such as Levosimendan, Milrinone, and Terbutaline—have emerged as potential candidates to rewire the molecular landscape of cancer cells [8,9,10]. By inhibiting phosphodiesterases (PDEs) or activating adrenergic signaling, these compounds elevate localized pools of the second messenger cyclic adenosine monophosphate (cAMP), which may, in turn, trigger the translocation of transport proteins, modulate lipid raft dynamics, and alter membrane potential [11,12]. This coordinated remodeling of the membrane may promote a state more permissive to 5-FU uptake, potentially enhancing intracellular delivery and metabolic stress by overcoming the biophysical barriers that define pseudo-resistance (Figure 2).

Here, we propose a hypothesis in which membrane-confined cAMP/PKA signaling acts as a central integrator of the cellular response to 5-FU, coordinating drug transport, membrane dynamics, redox balance, and downstream stress-response pathways. According to this conceptual model, the plasma membrane is not only the first physical barrier encountered by 5-FU, but also an active regulatory platform that may influence whether the drug efficiently enters the cell, accumulates at sufficient intracellular concentrations, and triggers cytotoxic stress. Importantly, several mechanisms discussed throughout this review—including cAMP/PKA-dependent regulation of transporter localization, lipid raft remodeling, and modulation of mitochondria-associated membrane (MAM) signaling—remain incompletely established in the specific context of 5-FU chemosensitization and should therefore be considered hypothesis-generating rather than definitive mechanistic conclusions. Nevertheless, accumulating evidence supports the broader concept that membrane-associated signaling and transport processes can influence therapeutic responsiveness and may represent actionable targets for pharmacological intervention.

This conceptual framework is anchored in experimental observations from a series of comparative studies conducted in urological cancer cell line models [8,9,10]. In these studies, human bladder cancer cells (UM-UC-5) and prostate cancer cells (PC-3) were systematically exposed to 5-FU alone and in combination with levosimendan, milrinone, and terbutaline under controlled in vitro conditions. Bladder cancer cells exhibited significantly greater sensitivity to these combination regimens, as evidenced by enhanced cytotoxic response, greater reductions in cell viability, and more pronounced induction of cell death markers compared with prostate cancer cells, which displayed consistently lower responsiveness across the tested combinations [8,9,10]. These experimentally characterized differences in drug response, obtained under robust and reproducible conditions, constitute the primary empirical motivation for the mechanistic framework developed in this review.

The differential response pattern observed across these models—greater sensitivity in UM-UC-5 cells, lower responsiveness in PC-3 cells—was not adequately explained by classical intracellular resistance mechanisms alone, suggesting the involvement of additional regulatory layers. This observation prompted the hypothesis that cancer cell models differ not only in their genetic or metabolic backgrounds, but also in the baseline functional organization and inducible capacity of their membrane-associated signaling and transport systems. The enhanced responsiveness of bladder cancer cells was interpreted as reflecting a more permissive or inducible membrane phenotype, in which cAMP/PKA-dependent remodeling maximizes solute carrier transport competence and lowers thresholds for stress-induced cell death [8]. Conversely, the lower responsiveness of prostate cancer cells was interpreted as reflecting a more restrictive membrane-regulated state, where transporter availability, lipid raft configuration, or stronger redox buffering may limit the intracellular impact of these combination regimens [9,10]. These experimentally grounded observations provide the empirical foundation upon which the mechanistic hypotheses developed throughout this review are constructed.

By placing the plasma membrane at the center of therapeutic response, this review aims to provide a conceptual and translational rationale for targeting membrane-regulated mechanisms as a strategy to overcome chemoresistance and improve the precision of combination therapies.

## 2. The Plasma Membrane as a Dynamic Regulatory Interface in Cancer

The regulatory and signaling functions of the plasma membrane are well established in cell biology [13,14].

What remains less understood, and therapeutically underexplored, is how cancer-specific alterations in membrane composition, organization, and signaling compartmentalization create functional states that are distinct from those of normal cells and that directly influence therapeutic response. The relevant question for the present framework is therefore not whether the membrane regulates cellular function—it clearly does—but rather how oncogenic reprogramming of membrane organization generates vulnerabilities that can be pharmacologically exploited to enhance chemosensitivity.

In cancer cells, alterations in lipid metabolism frequently reshape membrane composition in ways that differ qualitatively and quantitatively from non-transformed cells. Increased cholesterol content, altered sphingolipid profiles, and changes in phospholipid asymmetry have been documented across multiple tumor types [15,16] and are associated with reorganization of lipid microdomains [17], altered membrane fluidity [18], and redistribution of signaling receptors and transport proteins [19].

These cancer-specific changes are not simply quantitative variations in normal membrane biology but represent functional reprogramming that affects the spatial organization of signaling platforms, the surface availability of drug transporters, and the efficiency of receptor-coupled second messenger generation.

Particularly relevant to the present framework is the observation that cancer-associated membrane remodeling can create a state of functional pseudo-resistance to hydrophilic chemotherapeutic agents such as 5-Fluorouracil. Unlike lipophilic drugs that passively diffuse across the lipid bilayer, 5-FU depends critically on membrane transport proteins for intracellular entry. When cancer-specific alterations in membrane organization reduce the surface availability, lateral mobility, or functional coupling of these transporters—without necessarily altering their total cellular expression—the result is a membrane-imposed barrier to drug accumulation that is invisible to standard molecular profiling but functionally significant [20].

This concept of membrane-mediated pseudo-resistance distinguishes the present framework from classical resistance models focused on intracellular metabolism or genetic determinants and constitutes the central mechanistic contribution of this review.

Furthermore, the spatial compartmentalization of signaling pathways within cancer cell membranes differs from that of normal epithelia in ways that may determine the response to cAMP-modulating agents. Alterations in lipid raft composition, caveolin expression, and A-kinase anchoring protein (AKAP) scaffold organization can disrupt the normal spatial confinement of receptor-coupled signaling, reducing the precision and efficiency of downstream effector activation [19]. Continuous cycles of endocytosis and exocytosis regulate the surface availability of receptors and transporters, enabling rapid adaptation to environmental changes [21], and these processes are tightly coupled to signaling pathways and can modulate both the intensity and duration of cellular responses [22]. In this context, pharmacological elevation of submembrane cAMP by vasoregulatory agents may not simply amplify a normal signaling pathway but may partially restore a more permissive membrane organization that cancer-specific dysregulation has disrupted. This reframing positions cAMP-modulating chemosensitization not as an exogenous perturbation but as a functional restoration of membrane signaling competence—a conceptually distinct and therapeutically relevant perspective.

Understanding these cancer-specific features of membrane organization is therefore not a prerequisite for appreciating the regulatory role of membranes in general, but rather a necessary foundation for interpreting why pharmacological membrane remodeling may produce differential effects across tumor types [23,24,25], why pseudo-resistance emerges selectively in certain cancer models, and why integrated membrane phenotype profiling may provide more accurate predictions of therapeutic response than isolated molecular markers.

## 3. Membrane-Confined cAMP/PKA Signaling as a Regulatory Hub

### 3.1. Terbutaline: β_2_-Adrenergic Receptor-Mediated cAMP Generation at the Plasma Membrane

Terbutaline acts as a selective β_2_-adrenergic receptor (β_2_-AR) agonist, binding to the extracellular domain of this G protein-coupled receptor at the plasma membrane and inducing a conformational change that promotes coupling to the stimulatory Gs protein [26]. This interaction activates adenylate cyclase, catalyzing the conversion of ATP to cyclic AMP at the inner leaflet of the plasma membrane. The spatial confinement of this reaction to the submembrane compartment is mechanistically significant: cAMP generated at this interface preferentially activates PKA holoenzymes anchored in proximity to the receptor via A-kinase anchoring proteins, enabling rapid and spatially restricted phosphorylation of membrane-associated substrates [27,28]. Importantly, single-molecule microscopy studies have demonstrated that terbutaline induces immobilization of β_2_-AR-ligand complexes within the plasma membrane, suggesting that receptor confinement within specific membrane microdomains may be a functionally relevant consequence of agonist binding [27].

In cancer cells, β_2_-AR expression has been documented across multiple tumor types, including bladder and prostate cancer, where adrenergic signaling has been shown to influence proliferation, survival, and stress responses [29,30]. However, the downstream consequences of β_2_-AR activation are highly context-dependent. In certain tumor models, adrenergic stimulation has been associated with pro-survival signaling through PKA-dependent activation of cAMP response element binding (CREB) protein and anti-apoptotic pathways [31], while in others it has been linked to reduced proliferative capacity and enhanced sensitivity to cytotoxic stress [32]. This duality underscores the importance of cellular context—including receptor density, AKAP expression profile, and membrane organization—in determining the net functional outcome of β_2_-AR-mediated cAMP elevation [28]. In the context of the membrane phenotype model proposed here, terbutaline-induced cAMP generation may primarily influence membrane transport competence and lipid microdomain organization, rather than directly modulating survival signaling, although these effects are not mutually exclusive and require direct experimental dissection.

### 3.2. Milrinone: PDE3 Inhibition and Submembrane cAMP Accumulation

Milrinone exerts its pharmacological action through selective inhibition of phosphodiesterase-3 (PDE3), a dual-specificity enzyme that hydrolyzes both cAMP and cGMP, with a higher affinity for cAMP [33]. By preventing cAMP degradation, milrinone sustains elevated cyclic nucleotide concentrations in submembrane compartments, effectively prolonging and amplifying PKA activation in spatial proximity to the plasma membrane [28]. Two principal PDE3 isoforms are relevant in this context: PDE3A, which is predominantly expressed in cardiac myocytes and platelets, and PDE3B, which is more broadly expressed in adipose tissue, hepatocytes, and certain epithelial cell types [34]. The relative expression of these isoforms in bladder and prostate cancer cells has not been systematically characterized, representing an important knowledge gap that directly affects the predicted efficacy of milrinone-based chemosensitization.

In cancer cells, PDE3 activity has been reported to regulate cAMP-dependent processes including cell cycle progression, apoptotic signaling, and membrane protein trafficking [35,36]. Inhibition of PDE3 has been shown to induce growth arrest [37,38] and enhance apoptotic sensitivity [39] in selected tumor models, although these effects appear to be isoform- and context-dependent. Mechanistically, sustained submembrane cAMP accumulation following PDE3 inhibition may preferentially activate type II PKA holoenzymes associated with membrane-anchored AKAPs, leading to phosphorylation of targets including ion channels, cytoskeletal regulators, and potentially solute carrier transporters [28,40]. This spatial selectivity distinguishes PDE3 inhibition from global cAMP elevation and may be particularly relevant for the membrane-localized effects proposed in the current framework [28].

### 3.3. Levosimendan: Dual Mechanism and Mitochondrial Relevance

Levosimendan presents a pharmacologically distinct profile among the agents discussed here, combining PDE3 inhibitory activity with calcium-sensitizing effects on cardiac troponin C [41]. Its PDE3 inhibitory action operates through a mechanism analogous to milrinone, sustaining submembrane cAMP accumulation and prolonging PKA activation [28,41]. However, levosimendan additionally activates mitochondrial ATP-sensitive potassium channels (mitoKATP), an effect that has been associated with cytoprotection in cardiomyocytes but may have distinct implications in cancer cells [42].

In the oncological context, mitoKATP activation by levosimendan is of particular mechanistic interest. The opening of these channels influences mitochondrial membrane potential [43], electron transport chain activity [44], and Reactive Oxygen Species (ROS) generation [45], processes that are directly relevant to the mitochondria-associated membrane signaling and apoptotic threshold regulation discussed in previous sections of this review. Specifically, levosimendan-induced modulation of mitochondrial membrane dynamics may interact with MAM integrity and calcium flux, potentially lowering the threshold for mitochondrial outer membrane permeabilization under conditions of chemotherapeutic stress [46]. This dual mechanism, submembrane cAMP elevation combined with direct mitochondrial membrane modulation, may explain why levosimendan produces distinct effects compared with milrinone in certain experimental contexts, and suggests that its chemosensitizing potential may extend beyond cAMP-dependent membrane remodeling to encompass direct modulation of the mitochondrial apoptotic pathway [40]. These mechanistic distinctions remain largely hypothetical in the cancer context and require direct experimental validation.

### 3.4. Convergence on a Common Membrane-Associated Signaling Axis

Despite their pharmacological differences, terbutaline, milrinone, and levosimendan converge on a shared functional outcome: the elevation of cAMP concentrations within submembrane compartments and the consequent activation of membrane-anchored PKA signaling [28]. This spatial confinement is mechanistically critical, as it distinguishes localized membrane-associated cAMP signaling from the pleiotropic effects of global intracellular cAMP elevation [28].

The specificity of downstream effects is largely determined by the AKAP scaffold proteins that organize PKA in proximity to specific membrane substrates. Different AKAP isoforms direct PKA toward distinct targets—including ion channels, transporters, cytoskeletal proteins, and lipid-modifying enzymes—thereby enabling selective regulation of membrane function without globally disrupting intracellular signaling [28,40]. In this context, the composition and expression of the AKAP interactome in bladder versus prostate cancer cells may represent a critical determinant of differential drug response, and its systematic characterization would constitute a valuable experimental priority within the framework proposed here.

Importantly, while the convergence of these agents on cAMP/PKA signaling provides a unifying mechanistic rationale, their individual pharmacological profiles—receptor specificity, isoform selectivity, mitochondrial effects, and kinetic properties—introduce meaningful mechanistic heterogeneity that may translate into differential efficacy across tumor types and experimental conditions [28,41]. This heterogeneity should be explicitly addressed in future comparative studies rather than assumed to be pharmacologically equivalent.

## 4. cAMP-Driven Remodeling of Membrane Architecture and Transport Function

Lipid rafts provide a dynamic structural framework for the spatial organization of receptors and transporters, and serve as platforms for the assembly of localized cAMP/PKA signaling hubs [18,47].

Elevated cAMP levels and subsequent PKA activation can induce phosphorylation of cytoskeletal and membrane-associated proteins, leading to alterations in membrane architecture [48]. This process may result in the reorganization or partial disruption of lipid rafts, thereby modifying membrane fluidity and protein distribution [49].

Changes in membrane fluidity can significantly impact the function of transport proteins. For instance, the activity of ATP-dependent efflux pumps such as P-glycoprotein has been reported to be sensitive to membrane fluidity, although this effect appears to be context-dependent [50,51]. Importantly, these changes are not merely structural but have direct functional consequences, as membrane fluidity is a critical determinant of protein mobility, clustering, and interaction dynamics.

Beyond biophysical effects, cAMP/PKA signaling may regulate membrane transport through modulation of protein trafficking and post-translational modification, including the translocation and functional tuning of SLC transporters [52,53,54].

These mechanisms provide a rapid and reversible means of controlling membrane transport independently of transcriptional regulation, allowing cells to dynamically adapt to extracellular stimuli. In the context of chemotherapy, such regulation is particularly relevant for hydrophilic drugs such as 5-Fluorouracil, whose intracellular availability depends on membrane transport processes. By reshaping both the structural and functional properties of the plasma membrane, cAMP-elevating agents may influence drug uptake and retention. Consequently, this membrane-centered modulation emerges as a key mechanism contributing to the observed variability in therapeutic response across different cancer models.

## 5. The Transportome as a Dynamic Interface of Membrane-Regulated Drug Response

### 5.1. SLC Superfamily: Orchestrating 5-FU Influx and Localized Regulation

The intracellular bioavailability of 5-Fluorouracil is critically dependent on the coordinated activity of membrane transport systems, collectively referred to as the transportome. Among these, members of the Solute Carrier (SLC) superfamily play a central role by facilitating the translocation of hydrophilic molecules across the lipid bilayer [55]. In particular, equilibrative nucleoside transporters such as ENT1 (encoded by SLC29A1) are recognized as key determinants of 5-FU uptake, given the structural similarity of this drug to endogenous pyrimidines, which allows it to hijack these nucleoside pathways [56,57]. Consistent with this, both clinical and experimental evidence indicate that reduced ENT1 expression or activity is associated with decreased intracellular drug accumulation and diminished therapeutic efficacy [58,59,60]. Concentrative nucleoside transporters (CNTs), including SLC28A1, may further contribute to this process by mediating active uptake against concentration gradients, although their relative contribution appears to be context-dependent [61].

We hypothesize that elevated cAMP levels and subsequent PKA activation may induce phosphorylation of cytoskeletal and membrane-associated proteins, potentially leading to alterations in membrane architecture. This proposed sequence of events, including lipid raft reorganization, enhanced ENT1 membrane localization, and MAM sensitization lowering apoptotic thresholds, remains largely speculative in the specific context of 5-FU chemosensitization and should be interpreted as mechanistic hypotheses rather than established pathways.

Supporting evidence for cAMP/PKA-dependent transporter trafficking exists in other cellular contexts: PKA has been shown to regulate the membrane localization of several SLC transporters through phosphorylation-dependent recycling [62,63], and lipid raft integrity has been linked to nucleoside transporter function in non-cancer models [64,65]. However, direct experimental demonstration of PKA-driven ENT1 translocation in cancer cells exposed to 5-FU is currently lacking. Similarly, while MAM remodeling has been associated with altered apoptotic thresholds in response to cellular stress [66], its specific modulation by cAMP-elevating agents in the context of fluoropyrimidine therapy has not been directly demonstrated. Contradictory evidence also warrants consideration: cAMP/PKA signaling can exert pro-survival effects in certain cancer contexts, potentially opposing rather than enhancing chemosensitivity [67], and lipid raft disruption does not uniformly increase drug influx across tumor types [68]. These limitations underscore the need for systematic experimental validation before these mechanisms can be considered causally established.

In parallel, repurposed vasoregulatory agents such as levosimendan, milrinone and terbutaline may require membrane transport mechanisms to achieve effective intracellular accumulation, although the specific transport pathways involved remain incompletely characterized. Treatment outcomes may emerge from the integration of membrane structure, transport dynamics, and stress signaling, rather than from individual molecular targets.

Through modulation of cAMP signaling and membrane organization, these compounds may alter transporter localization, trafficking dynamics, or functional state, thereby indirectly affecting 5-FU uptake. In this context, changes in membrane architecture and signaling activity could create conditions that favor increased accessibility or efficiency of drug influx, without implying a direct or coordinated transport mechanism.

### 5.2. ABC Transporters: Efflux Barriers and Strategic Modulation

Opposing the role of SLC-mediated influx, ATP-binding cassette (ABC) transporters function as major determinants of drug efflux and are central to the development of multidrug resistance [69]. These transporters utilize ATP hydrolysis to actively export a wide range of compounds, thereby reducing intracellular drug concentrations [70,71]. Among them, P-glycoprotein (ABCB1) and multidrug resistance-associated protein 1 (MRP1/ABCC1) are particularly relevant in solid tumors, where their overexpression is frequently associated with poor therapeutic response [72].

The contribution of ABC transporters to differential drug response across cancer models is substantial, but highly context-dependent. In more resistant models, such as PC-3 and A549, increased expression and activity of efflux transporters may contribute to reduced intracellular accumulation of chemotherapeutic agents, thereby limiting cytotoxic efficacy [73,74]. However, in the case of 5-Fluorouracil, whose uptake and activity are strongly influenced by nucleoside transport systems and intracellular metabolism, the direct contribution of ABCB1-mediated efflux remains less clearly defined. This suggests that resistance in these models is unlikely to rely solely on classical efflux activity, but rather emerges from the integration of multiple regulatory mechanisms at the membrane and intracellular levels.

Within this framework, the interaction between efflux systems and repurposed agents such as Levosimendan and Milrinone may represent a relevant modulatory component. Although some vasodilatory compounds have been reported to interact with ABC transporters, current evidence does not robustly support a direct competitive inhibition of efflux pumps in the context of 5-Fluorouracil transport [75]. Rather than acting as direct inhibitors, these agents may influence transporter function indirectly by remodeling the biophysical landscape of the plasma membrane.

The functional balance between influx and efflux systems is therefore not only determined by transporter expression, but also by the membrane environment in which these proteins operate [76]. In this regard, cAMP-dependent signaling emerges as a potential regulator of membrane-associated processes, capable of modulating transporter activity, trafficking, and conformational dynamics [77]. Such effects may influence the efficiency of drug extrusion in a non-specific manner, contributing to increased intracellular retention without requiring direct inhibition of individual transporters [78]. Collectively, these observations support a model in which the transportome operates as a dynamic and signal-responsive membrane interface, integrating structural organization, intracellular signaling, and transporter function. Within this framework, cAMP-elevating agents may enhance the cellular response to 5-Fluorouracil not by directly targeting specific efflux pumps, but by optimizing the membrane’s ‘permissive state’ for drug retention and cytotoxic activity [79].

## 6. Redox Regulation and Organelle Membrane Crosstalk

Reactive oxygen species (ROS) play a central and context-dependent role in cancer biology, functioning both as signaling mediators and as drivers of cellular damage [80]. Importantly, ROS production is not uniformly distributed within the cell but is spatially organized across membrane compartments, including the plasma membrane, mitochondria, and endoplasmic reticulum [81]. This compartmentalization allows localized redox signaling within microdomains to modulate specific molecular targets and cellular processes [82].

In line with the membrane-confined signaling framework described above, cAMP/PKA activity has been implicated in the regulation of redox homeostasis. Through its effects on mitochondrial function, ion fluxes, and metabolic enzymes, cAMP signaling may influence both the generation and detoxification of ROS [83]. These effects are particularly relevant under chemotherapeutic stress, where redox imbalance can amplify drug-induced cytotoxicity [84].

MAMs, which represent specialized contact sites between the endoplasmic reticulum and mitochondria, have emerged as key platforms for the integration of redox, calcium and lipid signaling [85]. These membrane interfaces facilitate the transfer of Ca^2+^ from the endoplasmic reticulum to mitochondria, promoting mitochondrial metabolic activity but also sensitizing cells to oxidative stress [85]. Disruption or modulation of MAM integrity may therefore alter the threshold for mitochondrial dysfunction and apoptotic signaling [46].

In this context, localized ROS production at mitochondria and MAM interfaces can contribute to the induction of mitochondrial outer membrane permeabilization (MOMP), a critical step in apoptotic commitment [86]. The interplay between cAMP signaling, membrane organization, and transporter dynamics suggests the existence of distinct ‘membrane phenotypes’, emergent functional states that may influence therapeutic sensitivity beyond what single markers could predict [87].

In parallel, perturbations in endoplasmic reticulum homeostasis can activate the unfolded protein response (UPR), a membrane-associated adaptive pathway aimed at restoring proteostasis [88]. While initially cytoprotective, sustained UPR activation may shift toward pro-apoptotic signaling, particularly under conditions of unresolved stress [89]. The convergence of UPR activation, redox imbalance, and mitochondrial dysfunction highlights the role of membrane crosstalk in determining cell fate [89].

Within the context of chemotherapy, these interconnected processes are highly relevant for the activity of 5-Fluorouracil. By inducing replication stress and metabolic perturbations, 5-FU indirectly promotes oxidative stress, which is synergistically amplified by cAMP-dependent modulation of mitochondrial and membrane function. This synergy shifts the cellular balance from adaptive survival toward irreversible cell death.

Beyond their role in apoptotic commitment, redox processes may directly influence the membrane-dependent bioavailability of 5-Fluorouracil, particularly in the context of combination with cAMP-elevating vasoregulatory agents. Several mechanistic links between redox state and drug transport competence warrant explicit consideration. First, oxidative modification of membrane lipids, including lipid peroxidation and cholesterol oxidation, can alter membrane fluidity and microdomain organization, thereby affecting the lateral mobility, conformational dynamics, and functional state of embedded transport proteins such as ENT1 [90,91]. Under conditions of elevated ROS production, these biophysical changes may bidirectionally regulate transporter activity: moderate oxidative stress may enhance membrane fluidity and transiently increase transporter mobility, while severe or sustained oxidative damage may impair transporter function and reduce drug uptake [92]. The net effect on 5-FU bioavailability would therefore depend on the magnitude, duration, and subcellular localization of ROS generation induced by the combination regimen.

Second, ROS can directly modify transporter proteins through oxidation of cysteine residues and other redox-sensitive amino acids, altering their conformational state and functional activity independently of membrane biophysical changes [93]. Whether ENT1 or other SLC transporters relevant to 5-FU uptake contain functionally significant redox-sensitive residues has not been systematically investigated, representing a mechanistic gap of direct relevance to the proposed framework. Similarly, ABC efflux transporters, whose ATPase activity depends on the redox state of nucleotide-binding domains, may be differentially sensitive to oxidative modification, potentially reducing efflux capacity under conditions of chemotherapeutic oxidative stress and thereby contributing to increased intracellular drug retention [94].

Third, the metabolic activation of 5-FU to its cytotoxically active forms, including fluorodeoxyuridine monophosphate and fluorouridine triphosphate, involves enzymatic steps that are themselves sensitive to the intracellular redox environment [95]. Alterations in the NAD^+^/NADH ratio, glutathione availability, and thioredoxin system activity, which may be influenced by cAMP-dependent modulation of mitochondrial metabolism, could therefore indirectly affect the efficiency of 5-FU bioactivation and the accumulation of active metabolites at their intracellular targets [96,97].

Collectively, these considerations suggest that the redox state of the cancer cell—shaped by the combined actions of 5-FU-induced replication stress and cAMP-dependent mitochondrial modulation by vasoregulatory agents—may represent an additional and currently underappreciated determinant of intracellular drug bioavailability. Rather than operating exclusively downstream of drug accumulation, redox regulation may function as a concurrent modulator of transport competence, metabolic activation, and intracellular retention, integrating with the membrane phenotype framework in a manner that amplifies or attenuates the chemosensitizing effects of the proposed combination strategies. Direct experimental characterization of these redox–transport interactions, using approaches such as transporter activity assays under controlled oxidative conditions, redox-sensitive fluorescent probes, and metabolomic profiling of 5-FU activation intermediates, would be an important priority for future validation studies.

## 7. From Membrane Signaling to Cell Fate Decisions

The integration of membrane-confined cAMP/PKA signaling with chemotherapeutic stress ultimately converges on the regulation of cell fate decisions, particularly through coordinated effects on cell cycle progression and apoptotic commitment [98,99]. Rather than acting as independent processes, these outcomes reflect the cumulative impact of membrane remodeling, transport dynamics, and redox signaling described in previous sections.

Elevated cAMP levels have been associated with the modulation of key cell cycle regulators, including reduced expression of Cyclin D1 and increased activity of cyclin-dependent kinase inhibitors such as p21, resulting in cell cycle arrest at critical checkpoints including G1/S and G2/M [100,101]. In the context of 5-Fluorouracil treatment, this regulation may enhance the cytostatic response by limiting the ability of cancer cells to replicate damaged DNA and re-enter the proliferative cycle, thereby preventing the evasion of therapeutic pressure often associated with membrane-driven pseudo-resistance [102]. Importantly, such checkpoint enforcement is closely linked to upstream events at the membrane level, including drug uptake efficiency and signaling-dependent stress responses.

However, the transition from cytostatic arrest to irreversible cell death is governed by mechanisms that extend beyond cell cycle control. MOMP represents a critical threshold in this process, marking the point at which cells commit to apoptosis [103]. cAMP/PKA signaling may influence this transition by modulating the balance between pro- and anti-apoptotic proteins, including BAX and BCL-2, as well as through its effects on mitochondrial function and redox homeostasis [98].

In particular, the integration of redox signaling and calcium flux at MAMs may lower the threshold for mitochondrial dysfunction, facilitating cytochrome c release and downstream caspase activation. Under these conditions, membrane-associated signaling does not directly induce apoptosis but instead sensitizes cells to chemotherapeutic stress, effectively biasing the cellular response toward apoptotic commitment.

**Figure 3:**
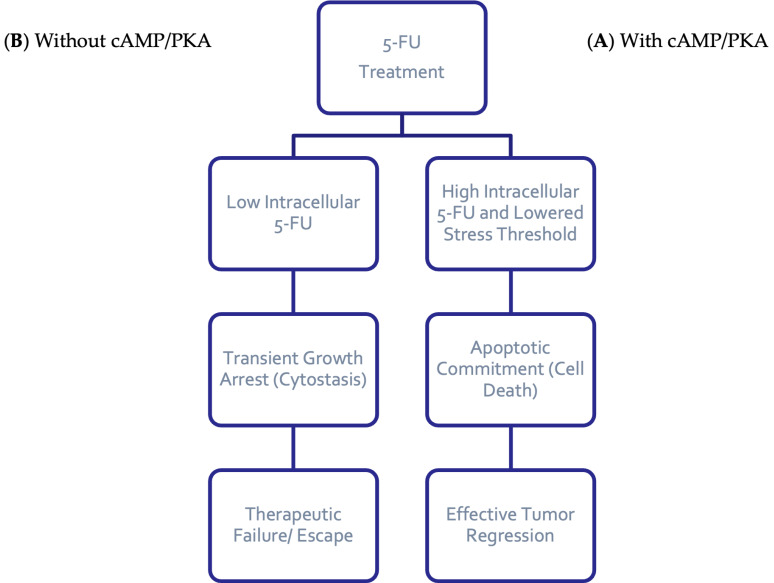
Decision map of membrane-regulated cell fate determines 5-FU disposition. The therapeutic outcome of 5-Fluorouracil treatment is governed by the membrane-signaling context. (**A**) In the presence of cAMP/PKA modulation, the membrane is remodeled to maximize 5-FU influx (via ENT1) and sensitize mitochondria-associated membranes (MAMs), lowering the apoptotic threshold and leading to irreversible cell death. (**B**) In the basal state (pseudo-resistance), restricted drug entry and high apoptotic thresholds limit the cellular response to transient growth arrest (cytostasis), often resulting in therapeutic failure and relapse.

Taken together, these observations support a model in which membrane-regulated signaling pathways do not act as primary cytotoxic drivers, but rather as modulators of cellular susceptibility. By coordinating drug availability, stress signaling, and checkpoint control, cAMP-dependent mechanisms may determine whether exposure to 5-Fluorouracil results in transient growth arrest or progression toward irreversible cell death, effectively priming the cellular landscape for enhanced therapeutic efficacy (Figure 3).

## 8. Differential Response in Bladder and Prostate Cancer Models

Variability in therapeutic response across cancer models remains a major challenge in the clinical application of chemotherapeutic agents, including 5-Fluorouracil [104]. While genetic and metabolic factors have traditionally been emphasized, increasing evidence suggests that differences in membrane organization and signaling may represent an additional and underappreciated layer of regulation [20,105].

The conceptual framework developed throughout this review is directly motivated by experimental observations obtained in a series of comparative studies conducted in urological cancer cell line models [8,9,10]. In these studies, human bladder cancer cells (UM-UC-5) and prostate cancer cells (PC-3) were systematically exposed to 5-FU alone and in combination with levosimendan, milrinone, and terbutaline under controlled in vitro conditions. Bladder cancer cells exhibited significantly greater sensitivity to these combination regimens, as evidenced by enhanced cytotoxic response, greater reductions in cell viability, and more pronounced induction of cell death markers compared with prostate cancer cells, which displayed consistently lower responsiveness across the tested combinations [8,10]. These experimentally characterized differences in drug response, obtained under robust and reproducible conditions, constitute the primary empirical motivation for the mechanistic framework proposed here and should be understood as the evidential foundation upon which the mechanistic hypotheses developed in this review are constructed.

Importantly, this differential response pattern was not adequately explained by classical intracellular resistance mechanisms alone, suggesting the involvement of additional regulatory layers. This observation prompted the hypothesis that these cancer models differ not only in their genetic or metabolic backgrounds, but also in the baseline functional organization and inducible capacity of their membrane-associated signaling and transport systems. The enhanced responsiveness of bladder cancer cells was interpreted as reflecting a more permissive or inducible membrane phenotype, in which cAMP/PKA-dependent remodeling maximizes solute carrier transport competence and lowers thresholds for stress-induced cell death [8]. Conversely, the lower responsiveness of prostate cancer cells was interpreted as reflecting a more restrictive membrane-regulated state, where transporter availability, lipid raft configuration, or stronger redox buffering may limit the intracellular impact of these combination regimens [8,10].

In particular, the efficiency of drug uptake through solute carrier transporters and the activity of efflux systems such as ABC transporters may vary significantly between cell types, influencing intracellular drug availability [106,107]. However, as discussed in previous sections, these systems do not operate in isolation but are dynamically regulated by membrane-associated signaling networks. Differences in transporter function may arise not only from expression levels but also from variations in membrane architecture and signaling context, effectively redefining the functional transportome of each cancer type [77].

Additionally, heterogeneity in redox homeostasis and mitochondrial function may further contribute to differential sensitivity [108,109]. Variations in the capacity to buffer oxidative stress or to engage apoptotic pathways can influence the threshold at which chemotherapeutic stress transitions from cytostatic arrest to cell death [110]. In this regard, differences in mitochondria–ER crosstalk and in the regulation of calcium and ROS signaling may play a central role in determining the apoptotic threshold of bladder versus prostate cancer cells, dictated by their distinct capacities to manage localized ROS signals.

Within this framework, the responsiveness of different cancer models to cAMP-modulating agents in combination with 5-Fluorouracil may reflect their underlying membrane signaling landscape. Rather than being solely determined by individual molecular targets, treatment outcomes may emerge from the integration of membrane structure, transport dynamics, and stress signaling pathways.

This perspective provides a conceptual basis to interpret heterogeneous responses across tumor types, suggesting that distinct membrane phenotypes, defined by the integration of signaling, transport, and structural properties, and grounded in experimentally observed differential drug sensitivity, may act as key determinants of therapeutic responsiveness to 5-FU-based combination strategies.

## 9. Integration with Canonical 5-FU Resistance Pathways

The membrane-centric model proposed in this review does not contest the established role of intracellular resistance mechanisms in determining 5-FU therapeutic failure. Rather, it proposes that membrane-regulated processes constitute an additional and functionally upstream layer of regulation that modulates the intracellular context in which canonical resistance pathways operate. Reconciling these two levels of analysis is essential for a complete mechanistic understanding of chemoresistance and for the rational design of combination strategies.

Among the best-characterized determinants of 5-FU resistance, thymidylate synthase upregulation represents perhaps the most clinically relevant [111]. Elevated thymidylate synthase (TYMS) expression reduces the efficacy of fluorodeoxyuridine monophosphate-mediated enzyme inhibition, a central mechanism of 5-FU cytotoxicity, by increasing the pool of available enzyme and thereby titrating out the drug effect [112]. Critically, however, TYMS-mediated resistance presupposes that sufficient intracellular drug has been converted to its active metabolites. If membrane-dependent uptake is rate-limiting, as proposed in the pseudo-resistance framework developed here, then TYMS upregulation may represent a secondary resistance layer that becomes dominant only once the primary transport barrier has been overcome. In this sense, membrane phenotype and TYMS status may interact sequentially rather than independently, with transport competence determining the magnitude of intracellular drug exposure and TYMS activity determining the efficiency of its downstream cytotoxic conversion.

Dihydropyrimidine dehydrogenase (DPD) overexpression presents a partially distinct relationship with membrane regulation. As the rate-limiting enzyme of 5-FU catabolism, DPD activity determines the fraction of intracellular drug available for anabolic activation versus degradation [113]. High DPD activity effectively shortens the intracellular half-life of 5-FU, reducing the window during which active metabolites can exert cytotoxic effects [114]. In tumors where both membrane-limited uptake and high DPD activity coexist, the combined effect may produce profound therapeutic failure that neither mechanism alone would fully explain. Conversely, enhancing membrane permeability through cAMP-dependent remodeling in a high-DPD context might increase drug flux sufficiently to saturate catabolic capacity, partially restoring therapeutic efficacy. This interaction has not been directly tested but represents a tractable experimental hypothesis with clear translational relevance.

Mismatch repair (MMR) deficiency introduces a further dimension of complexity. Loss of MMR function impairs the recognition of 5-FU-induced DNA mismatches, reducing the apoptotic signaling that would normally follow drug-induced replication stress [115]. Within the membrane-centered framework, MMR deficiency can be understood as a downstream resistance mechanism that operates independently of drug uptake but determines the cellular response once the drug has reached its nuclear targets. Importantly, cAMP-dependent modulation of mitochondrial apoptotic thresholds, as discussed in previous sections, may partially circumvent MMR-dependent resistance by engaging apoptotic pathways through mitochondrial rather than DNA damage-driven signaling [98,116]. Whether this alternative apoptotic route is sufficient to overcome MMR deficiency-associated resistance remains an open and experimentally addressable question.

Beyond these three canonical mechanisms, additional resistance determinants, including reduced thymidine kinase activity [117], altered folate metabolism affecting 5,10-methylenetetrahydrofolate availability [118], and autophagy-mediated stress adaptation [119], may interact with membrane phenotype in ways that are currently unexplored. The common thread across these interactions is that membrane-regulated drug availability sets the quantitative input to all downstream resistance mechanisms. A tumor with a restrictive membrane phenotype may appear resistant regardless of its intracellular biochemical profile, simply because insufficient drug accumulates to engage any cytotoxic pathway at a therapeutically meaningful level [120].

This integrative perspective suggests that biomarker strategies and combination approaches should ideally account for both membrane phenotype and canonical resistance status. Tumors with permissive membrane phenotypes and low TYMS or DPD activity may represent the population most likely to benefit from cAMP-modulating chemosensitization. Conversely, tumors with restrictive membrane phenotypes combined with high TYMS expression or MMR deficiency may require multi-layered interventions that simultaneously address transport barriers, metabolic resistance, and downstream apoptotic competence. Recognizing these interactions does not diminish the relevance of the membrane model but rather clarifies the patient subpopulations in which it is most likely to exert meaningful therapeutic impact.

## 10. Translational Implications and Biomarkers

### 10.1. Biomarker Framework and Patient Stratification

The membrane-centric framework proposed in this review has important translational implications for the development of more precise and functionally informed strategies to predict response to 5-Fluorouracil-based therapies. In current clinical and experimental settings, therapeutic stratification is often guided by isolated molecular markers, such as the expression of enzymes involved in fluoropyrimidine metabolism, individual transporter levels, or selected resistance-associated genes. Although informative, these markers may not fully capture the dynamic and spatially organized processes that regulate drug entry, intracellular retention, stress signaling, and apoptotic commitment. The model proposed here suggests that treatment response may be more accurately predicted by integrated functional profiles rather than by single biomarkers considered in isolation.

Within this context, a multi-parameter biomarker strategy should include three complementary dimensions: membrane-confined signaling capacity, drug transport competence, and membrane structural organization. The first dimension relates to the ability of tumor cells to generate and sustain localized cAMP signaling. Key determinants may include the expression and enzymatic activity of phosphodiesterase-3 isoforms, particularly PDE3A and PDE3B, which regulate local cAMP degradation, as well as the density and functional coupling of β_2_-adrenergic receptors, which determine the capacity of tumor cells to respond to adrenergic stimulation and activate downstream cAMP/PKA signaling [36,121]. In addition, the expression or spatial organization of A-kinase anchoring proteins may be relevant, as these scaffolding proteins contribute to the compartmentalization of PKA signaling within specific membrane microdomains.

The second dimension concerns the functional state of membrane transport systems. For hydrophilic drugs such as 5-Fluorouracil, intracellular bioavailability depends not only on extracellular drug exposure but also on the balance between influx and efflux mechanisms. Solute carrier transporters, particularly equilibrative nucleoside transporter 1, encoded by SLC29A1, represent relevant candidate biomarkers because of their role in nucleoside analogue uptake. However, total ENT1 expression alone may be insufficient. Its membrane localization, trafficking dynamics, and functional activity may be equally important in determining effective 5-FU uptake. Similarly, the activity of ATP-binding cassette transporters, including ABCB1 and potentially other multidrug resistance-associated transporters, may influence intracellular drug retention, although their contribution to 5-FU response is likely context-dependent. Therefore, the clinically relevant parameter may be the functional ratio between influx capacity and efflux pressure rather than the isolated expression of any single transporter.

The third dimension involves membrane architecture and microdomain organization. Lipid raft integrity, cholesterol content, membrane fluidity, and the spatial distribution of receptors and transporters may strongly influence both signaling efficiency and drug transport. Tumors with highly rigid or structurally restrictive membranes may be less permissive to transporter mobility and drug uptake, contributing to a state of membrane-mediated pseudo-resistance. Conversely, tumors in which membrane organization can be dynamically remodeled by cAMP-modulating agents may become more susceptible to 5-FU accumulation and downstream cytotoxicity. In this sense, lipid raft markers, membrane cholesterol distribution, caveolin expression, and transporter co-localization within specific membrane domains may provide useful experimental readouts of membrane phenotype.

Importantly, these signaling, transport, and structural features should not be interpreted independently. Their integration may define distinct functional “membrane phenotypes” with direct relevance for therapeutic response. A permissive membrane phenotype could be characterized by preserved β_2_-adrenergic receptor responsiveness, moderate or low PDE3-mediated cAMP degradation, efficient ENT1 membrane localization, favourable influx-to-efflux balance, adaptable lipid raft organization, and a low threshold for mitochondrial apoptotic activation. Such tumors may be particularly sensitive to combination strategies involving 5-FU and cAMP-modulating agents such as levosimendan, milrinone, or terbutaline. In contrast, a restrictive membrane phenotype may be characterized by impaired cAMP compartmentalization, reduced transporter recruitment to the membrane, high efflux activity, rigid lipid microdomains, and increased resistance to mitochondrial stress, thereby limiting the effectiveness of these combinations.

This conceptual framework also has implications for the interpretation of differential sensitivity across cancer models. The greater responsiveness observed in bladder cancer cells compared with prostate cancer models may reflect differences in membrane phenotype rather than differences in a single canonical oncogenic pathway. Bladder cancer cells may present a more permissive configuration, with membrane-associated signaling and transport systems that remain responsive to pharmacological remodeling. Prostate cancer cells, by contrast, may display a more restrictive phenotype, potentially associated with altered transporter activity, different lipid raft organization, stronger adaptive redox buffering, or a higher apoptotic threshold. This interpretation should be experimentally validated, but it provides a mechanistic rationale for why the same combination strategy may produce divergent outcomes across tumor types.

From a translational perspective, the proposed membrane phenotype could be evaluated using a stepwise biomarker panel. At the molecular level, candidate markers would include PDE3A/PDE3B, ADRB2, AKAP isoforms, SLC29A1/ENT1, SLC28A1/CNT1, ABCB1, ABCC1, caveolin-1, flotillin-1, and selected markers of lipid raft organization. At the functional level, relevant assays would include intracellular cAMP quantification, PKA activity, 5-FU uptake and retention assays, transporter localization by immunofluorescence or membrane fractionation, cholesterol depletion or lipid raft disruption experiments, and efflux activity assays. At the cellular response level, this panel could be complemented by measurements of ROS generation, mitochondrial membrane potential, BAX/BCL-2 ratio, caspase activation, and cell-cycle arrest markers such as Cyclin D1 and p21. Together, these readouts would allow the classification of tumor models according to their capacity to undergo membrane-regulated sensitization to 5-FU.

This approach could also support the design of preclinical validation studies. First, basal membrane phenotypes could be compared between sensitive and resistant tumor cell lines. Second, cells could be treated with 5-FU alone or in combination with levosimendan, milrinone, or terbutaline to determine whether cAMP-modulating agents enhance ENT1 membrane localization, increase intracellular 5-FU accumulation, reduce efflux activity, or lower the apoptotic threshold. Third, mechanistic inhibition experiments could be performed using PKA inhibitors, β_2_-adrenergic receptor blockade, PDE3 modulation, lipid raft disruption, or transporter silencing to determine whether the proposed pathway is causally involved in the observed chemosensitization. Finally, validation in three-dimensional tumor spheroids, patient-derived organoids, or ex vivo tumor explants would strengthen the clinical relevance of this model.

Clinically, the development of membrane phenotype biomarkers may contribute to more personalized combination strategies. Instead of treating all patients with uniform fluoropyrimidine-based regimens, tumors could be stratified according to their predicted capacity for membrane remodeling and enhanced drug uptake. Patients with permissive or inducible membrane phenotypes may be more likely to benefit from the addition of repurposed cAMP-modulating agents, whereas patients with restrictive phenotypes may require alternative strategies, such as direct transporter modulation, lipid-targeting approaches, or combinations that bypass membrane-dependent uptake mechanisms. This would represent a shift from static molecular stratification toward a more functional and systems-level approach to biomarker development.

Importantly, this translational model should be interpreted as hypothesis-generating rather than immediately clinically actionable. The biomarkers proposed here require systematic validation across multiple tumor types, standardized assays, and clinically relevant models. Moreover, the pharmacological use of cAMP-modulating agents in oncology must consider dose, schedule, cardiovascular safety, tumor context, and potential off-target effects. Nevertheless, because levosimendan, milrinone, and terbutaline are already clinically characterized drugs, their repurposing may offer a pragmatic route for translational development if supported by robust mechanistic and preclinical evidence.

Collectively, these considerations highlight the need to move beyond single-marker predictors of 5-FU response and toward integrated, functional biomarker frameworks. By combining membrane signaling, transporter activity, lipid microdomain organization, redox state, and apoptotic competence, the membrane phenotype concept may provide a more biologically realistic basis for predicting therapeutic sensitivity. This strategy has the potential to refine patient stratification, identify tumors vulnerable to cAMP-based chemosensitization, and guide the rational development of combination therapies aimed at overcoming membrane-mediated chemoresistance (Table 1).

### 10.2. Clinical Feasibility, Therapeutic Window, and Safety Considerations

A critical prerequisite for the translational development of levosimendan, milrinone, and terbutaline as chemosensitizing agents is the demonstration that membrane-modulating exposures can be achieved within a clinically acceptable therapeutic window. Each of these compounds carries a well-characterized cardiovascular pharmacology that must be rigorously considered before oncological repurposing can be responsibly proposed.

Levosimendan is currently approved for acute decompensated heart failure and is administered as a short-term intravenous infusion, typically at 0.05–0.2 µg/kg/min, with plasma concentrations in the low nanomolar range [122]. Its principal dose-limiting effects include hypotension, tachycardia, and arrhythmia, largely attributable to vasodilation and PDE3 inhibition in vascular smooth muscle [123]. Whether the concentrations required to achieve meaningful cAMP elevation and membrane remodeling in tumor cells fall within or below the cardiovascular exposure range remains unknown. This represents a fundamental translational gap: preclinical studies using supraphysiological concentrations in cell culture models may not translate to clinically achievable exposures, and future studies should explicitly report concentration-response relationships in the context of human pharmacokinetic data.

Milrinone, another PDE3 inhibitor used in acute cardiac failure, presents a similar challenge. Although short-term intravenous administration is well tolerated hemodynamically in monitored settings, chronic or repeated use has been associated with increased mortality in heart failure patients, raising important questions about scheduling and cumulative exposure in an oncological context [124]. Intermittent or pulse-dosing strategies, potentially timed to coincide with chemotherapy cycles, may represent a more feasible approach, but would require dedicated pharmacodynamic modeling and careful cardiac monitoring.

Terbutaline, a selective β_2_-adrenergic receptor agonist used clinically as a bronchodilator, presents a somewhat more favorable safety profile at standard doses, with principal adverse effects including tachycardia, tremor, and hypokalemia [124]. Its oral bioavailability and established use in chronic respiratory disease make it potentially more tractable for repurposing. However, β_2_-adrenergic signaling in cancer cells is itself not without complexity: preclinical evidence suggests that adrenergic stimulation can promote tumor progression in certain contexts, including enhanced invasiveness and immunosuppression [125,126], a concern that must be carefully weighed against any putative chemosensitizing benefit.

Importantly, the oncological patient population may present additional cardiovascular vulnerabilities. Patients receiving fluoropyrimidine-based chemotherapy are themselves at elevated risk of cardiotoxicity, including coronary vasospasm, arrhythmia, and myocardial dysfunction [127]. The potential compounding of these effects by PDE3 inhibitors or β_2_-adrenergic agonists represents a clinically meaningful safety concern that must be prospectively addressed in any translational development program.

Several mitigation strategies may be worth exploring in this context. Local or tumor-directed delivery approaches, where anatomically feasible, could reduce systemic cardiovascular exposure while maintaining effective intratumoral concentrations. Lower-dose or intermittent regimens designed to achieve submaximal cAMP elevation may preserve chemosensitizing activity with reduced hemodynamic impact. Rigorous cardiovascular risk stratification would be essential for patient selection in any future clinical trial. Finally, ex vivo organoid or tumor explant models using pharmacokinetically relevant drug concentrations would allow the pharmacodynamic relationship to be evaluated in a translationally grounded manner prior to human studies.

We acknowledge that, at present, the evidence base does not allow a definitive conclusion on whether membrane-modulating cAMP elevations can be safely and reproducibly achieved in cancer patients at therapeutically meaningful concentrations. This remains a fundamental and unresolved translational question. The framework proposed here should therefore be interpreted strictly as hypothesis-generating, identifying a pharmacological rationale for repurposing rather than constituting a clinically actionable recommendation. Pharmacokinetic–pharmacodynamic characterization, cardiovascular safety assessment, and dose-finding studies in relevant preclinical models represent necessary and non-negotiable steps before any clinical translation can be responsibly considered. Because levosimendan, milrinone, and terbutaline are already clinically characterized compounds with established safety profiles in their approved indications, their repurposing may nonetheless offer a pragmatic and accelerated route to translational development if supported by robust mechanistic and preclinical evidence.

## 11. Conclusions

The plasma membrane should no longer be viewed merely as a passive boundary separating the extracellular and intracellular environments. Instead, it emerges as a dynamic regulatory interface capable of integrating signaling, transport, redox balance, organelle communication, and stress-response pathways to shape the final cellular outcome of chemotherapy. In the context of 5-Fluorouracil, this membrane-centered perspective provides a mechanistic explanation for how drug availability, intracellular retention, and apoptotic commitment may be regulated before the drug reaches its canonical intracellular targets. Membrane-confined cAMP/PKA signaling appears to be a critical component of this process, acting as a molecular bridge between receptor activation, transporter function, lipid microdomain remodeling, redox homeostasis, and mitochondrial vulnerability. By coordinating these processes, cAMP-modulating agents such as levosimendan, milrinone, and terbutaline may shift cancer cells from a restrictive or pseudo-resistant state toward a more permissive membrane phenotype, thereby improving 5-FU uptake and enhancing therapeutic response.

Critically, the membrane phenotype framework proposed here does not operate in isolation from established resistance biology. Rather, membrane-regulated drug availability constitutes a functionally upstream layer that determines the intracellular context in which canonical resistance mechanisms—including thymidylate synthase upregulation, dihydropyrimidine dehydrogenase overexpression, and mismatch repair deficiency—ultimately exert their effects. Recognizing this hierarchical relationship provides a more complete mechanistic basis for understanding why some tumors fail to respond to fluoropyrimidine-based therapy even in the absence of classical resistance markers, and identifies membrane phenotype stratification as a potentially valuable complement to existing molecular profiling approaches.

This framework has important implications for drug repurposing and personalized oncology. Rather than focusing exclusively on single intracellular targets or isolated genetic markers, therapeutic response may be better understood through integrated functional profiles that combine membrane signaling capacity, transporter activity, lipid raft organization, redox state, and apoptotic competence. The concept of a “membrane phenotype” may therefore support the development of new biomarker panels, guide patient stratification, and inform rational combination strategies designed to overcome chemoresistance.

Importantly, the clinical translation of this framework requires careful consideration of the cardiovascular pharmacology of the proposed agents. Levosimendan, milrinone, and terbutaline carry well-characterized hemodynamic profiles and dose-limiting toxicities that must be rigorously addressed before oncological repurposing can be responsibly pursued. Pharmacokinetic–pharmacodynamic characterization, cardiovascular safety assessment, and dose-finding studies in clinically relevant preclinical models represent necessary prerequisites. Their established clinical profiles may nonetheless offer a pragmatic route to translational development if supported by robust mechanistic and preclinical evidence.

Beyond 5-Fluorouracil-based chemotherapy, the broader relevance of this model may extend to other anticancer drugs whose efficacy depends on membrane transport, receptor signaling, or intracellular accumulation, and may have potential value across other fields of medicine in which membrane organization and compartmentalized signaling are central determinants of disease and treatment response.

Future studies should validate this hypothesis using integrated experimental models, including comparative cell-line panels, three-dimensional tumor spheroids, patient-derived organoids, and ex vivo tumor explants. Particular attention should be given to functional biomarkers of membrane remodeling, such as ENT1 localization, ABC transporter activity, lipid raft integrity, intracellular cAMP dynamics, mitochondrial stress, and apoptotic threshold. If validated, this approach could contribute to a new generation of personalized combination therapies in which the membrane is not simply a barrier to be crossed, but a therapeutic interface to be functionally reprogrammed. Ultimately, targeting the membrane phenotype may provide a powerful strategy to overcome chemoresistance by reshaping the cellular interface that governs drug availability, signaling integration, and cell fate decisions.

## Figures and Tables

**Table 1:** Candidate components of a membrane phenotype biomarker panel for predicting response to 5-Fluorouracil and cAMP-modulating combination strategies.

Biomarker Domain	Candidate Markers/Readouts	Translational Relevance
cAMP signaling	PDE3A/PDE3B, ADRB2, AKAPs, intracellular cAMP, PKA activity	Defines capacity for compartmentalized cAMP/PKA signaling.
Drug influx	ENT1/SLC29A1, CNT1/SLC28A1, transporter membrane localization	Predicts 5-FU uptake capacity.
Drug efflux	ABCB1, ABCC1, efflux activity assays	Estimates resistance-associated drug extrusion.
Membrane architecture	Lipid rafts, cholesterol content, caveolin-1, flotillin-1, membrane fluidity	Defines permissive or restrictive membrane organization.
Stress response	ROS, mitochondrial membrane potential, MAM integrity	Indicates vulnerability to treatment-induced stress.
Cell fate	BAX/BCL-2, caspase activation, Cyclin D1, p21	Links membrane remodeling to cytostasis or apoptosis.

Abbreviations: 5-FU, 5-Fluorouracil; ABCB1, ATP-binding cassette subfamily B member 1; ABCC1, ATP-binding cassette subfamily C member 1; ADRB2, beta-2 adrenergic receptor; AKAPs, A-kinase anchoring proteins; BAX, BCL2-associated X; BCL-2, B-cell lymphoma 2; cAMP, cyclic adenosine monophosphate; CNT1, concentrative nucleoside transporter 1; ENT1, equilibrative nucleoside transporter 1; MAM, mitochondria-associated membrane; PDE3, phosphodiesterase-3; PKA, protein kinase A; ROS, reactive oxygen species.

## Data Availability

Not applicable.
